# Construction of possible integrated predictive index based on *EGFR* and *ANXA3* polymorphisms for chemotherapy response in fluoropyrimidine-treated Japanese gastric cancer patients using a bioinformatic method

**DOI:** 10.1186/s12885-015-1721-z

**Published:** 2015-10-16

**Authors:** Hiro Takahashi, Nahoko Kaniwa, Yoshiro Saito, Kimie Sai, Tetsuya Hamaguchi, Kuniaki Shirao, Yasuhiro Shimada, Yasuhiro Matsumura, Atsushi Ohtsu, Takayuki Yoshino, Toshihiko Doi, Anna Takahashi, Yoko Odaka, Misuzu Okuyama, Jun-ichi Sawada, Hiromi Sakamoto, Teruhiko Yoshida

**Affiliations:** 1 Graduate School of Horticulture, Chiba University, 648 Matsudo, Matsudo, Chiba 271-8510 Japan; 2 Plant Biology Research Center, Chubu University, Matsumoto-cho 1200, Kasugai, Aichi 487-8501 Japan; 3 Division of Genetics, National Cancer Center Research Institute, 5-1-1 Tsukiji, Chuo-ku, Tokyo 104-0045 Japan; 4 Division of Medicinal Safety Science, National Institute of Health Sciences, 1-18-1 Kamiyoga, Setagaya-ku, Tokyo 158-8501 Japan; 5 Gastrointestinal Medical Oncology Division, National Cancer Center Hospital, 5-1-1 Tsukiji, Chuo-ku, Tokyo 104-0045 Japan; 6 Division of Developmental Therapeutics, Research Center for Innovative Oncology, National Cancer Center Hospital East, 6-5-1, Kashiwanoha, Kashiwa, Chiba 277-8577 Japan; 7 Department of Gastrointestinal Oncology, National Cancer Center Hospital East, 6-5-1, Kashiwanoha, Kashiwa, Chiba 277-8577 Japan; 8 Division of Functional Biochemistry and Genomics, National Institute of Health Sciences, 1-18-1 Kamiyoga, Setagaya-ku, Tokyo 158-8501 Japan; 9 Present address: Pharmaceutical and Medical Devices Agency, Shinkasumigaseki-building, 3-3-2 Kasumigaseki, Chiyoda-ku, Tokyo 100-0013 Japan

**Keywords:** Single nucleotide polymorphisms, Bioinformatics, Gastric cancer, Genome-wide association study, Fluoropyrimidine]

## Abstract

**Background:**

Variability in drug response between individual patients is a serious concern in medicine. To identify single-nucleotide polymorphisms (SNPs) related to drug response variability, many genome-wide association studies have been conducted.

**Methods:**

We previously applied a knowledge-based bioinformatic approach to a pharmacogenomics study in which 119 fluoropyrimidine-treated gastric cancer patients were genotyped at 109,365 SNPs using the Illumina Human-1 BeadChip. We identified the SNP rs2293347 in the human epidermal growth factor receptor (*EGFR*) gene as a novel genetic factor related to chemotherapeutic response. In the present study, we reanalyzed these hypothesis-free genomic data using extended knowledge.

**Results:**

We identified rs2867461 in annexin A3 (*ANXA3*) gene as another candidate. Using logistic regression, we confirmed that the performance of the rs2867461 + rs2293347 model was superior to those of the single factor models. Furthermore, we propose a novel integrated predictive index (iEA) based on these two polymorphisms in *EGFR* and *ANXA3*. The *p* value for iEA was 1.47 × 10^−8^ by Fisher’s exact test. Recent studies showed that the mutations in *EGFR* is associated with high expression of dihydropyrimidine dehydrogenase, which is an inactivating and rate-limiting enzyme for fluoropyrimidine, and suggested that the combination of chemotherapy with fluoropyrimidine and EGFR-targeting agents is effective against EGFR-overexpressing gastric tumors, while *ANXA3* overexpression confers resistance to tyrosine kinase inhibitors targeting the *EGFR* pathway.

**Conclusions:**

These results suggest that the iEA index or a combination of polymorphisms in *EGFR* and *ANXA3* may serve as predictive factors of drug response, and therefore could be useful for optimal selection of chemotherapy regimens.

**Electronic supplementary material:**

The online version of this article (doi:10.1186/s12885-015-1721-z) contains supplementary material, which is available to authorized users.

## Background

Inter-individual variation in drug response is clinically expected, but relatively difficult to predict [1, 2]. Chemotherapy, in particular, is plagued by highly variable response rates as well as significant toxicity [1]. Genetic variation is an important cause of inter-individual variability in drug response. Dihydropyrimidine dehydrogenase (DPD), an enzyme encoded by the *DPYD* gene, plays a key role in the adverse effects of fluoropyrimidine treatment: it participates in the catabolism of fluoropyrimidines, such as 5-fluorouracil (5-FU) and its prodrugs capecitabine and S-1 (trade name TS-1, the 5-fluorouracil derivative developed by Tetsuhiko Shirasaka). DPD is an inactivating and rate-limiting enzyme for 5-FU, which is used in various chemotherapeutic regimens to treat gastrointestinal, breast, and head/neck cancers [3]. The antitumor effect of 5-FU is due to its intracellular conversion into antiproliferative nucleotides via anabolic pathways. DPD affects 5-FU availability by rapidly degrading it to 5,6-dihydrofluorouracil (DHFU) [4]. 5-FU catabolism occurs in various tissues including tumors, but is most active in the liver [5, 6].

Wide variability in DPD activity (8- to 21-fold) was shown in Caucasians, and 3–5 % of Caucasians had reduced DPD activity [7, 8]. To date, at least 68 variant *DPYD* alleles exerting various effects on DPD activity have been reported [3, 9–13]. Of these alleles, the splice site polymorphism IVS14 + 1G>A, which causes skipping of exon 14, is occasionally detected in Northern Europeans with an allele frequency of 0.01–0.02 [9]. Of the patients with a 5-FU-associated grade 3 or 4 adverse event, 24–28 % are heterozygous or homozygous for the IVS14 + 1G>A single nucleotide polymorphism (SNP) [9]. This SNP, however, has not been reported in Japanese or African-American populations [3], and therefore this SNP is not predictive of antitumor effect.

A genome-wide association study (GWAS) is an examination of many common genetic variants in different individuals to determine whether a particular variant is associated with a trait. GWAS using hypothesis-free genomic data is a powerful approach to identify common genetic variants between patients. However, multiple testing problems are a limitation of this approach. We addressed this issue in previous reports by proposing a combined method consisting of a knowledge-based algorithm, two stages of screening, and permutation test to identify significant SNPs [14]. The usability of our combined method was confirmed by applying it into another dataset [15]. In general, the objective of statistical or bioinformatics analysis is the enrichment of important information from a large dataset [16–25]. The use of a knowledge-based algorithm is not a novel concept, but is both practical and useful [26–36]. In the previous study, we applied our combined method to data from gastric cancer patients treated with fluoropyrimidine [14]. We found that rs2293347 in the human epidermal growth factor receptor (*EGFR*) is a candidate SNP related to chemotherapeutic response and antitumor effect. Nonetheless, the comprehensiveness of the method was limited.

In the present study, to achieve a more comprehensive analysis, we applied our combined method based on an extended knowledge to the dataset of the previous study. Using this approach, we identified rs2867461 in annexin A3 (*ANXA3*) gene related to the chemotherapeutic response as a novel candidate SNP. Based on discovery of this SNP, we proposed an integrated predictive index based on these two polymorphisms in *EGFR* and *ANXA3* and tested performance of this index. Furthermore, we constructed an *EGFR* and *ANXA3* relation model related to fluoropyrimidine resistance, according to the literature.

## Methods

### Ethics statement

This study was conducted according to the principles expressed in the Declaration of Helsinki. The ethics committees of the National Cancer Center and National Institute of Health Sciences, Japan, approved the study protocol. All patients provided written informed consent.

### Preparation of hypothesis-free genomic data on gastric cancer patients treated with fluoropyrimidine

This study was performed within the framework of the Millennium Genome Project in Japan. A total of 128 Japanese fluoropyrimidine-naïve gastric cancer patients at the National Cancer Center Hospital and National Cancer Center Hospital East were included in the study. DNA samples were extracted from peripheral blood mononuclear cells and 109,365 SNPs were genotyped using the Illumina Human-1 BeadChip. We further restricted our analysis to 119 of the 128 patients whose chemotherapeutic responses were evaluated using Response Evaluation Criteria in Solid Tumors (RECIST). Among the 119 gastric cancer patients, 58 patients were treated with S-1, 27 patients were treated with 5-FU/methotrexate (5-FU/MTX), 33 patients were treated with high-dose 5-FU, and 1 patient was treated with low-dose 5-FU. We defined the 58 patients treated with S-1 as the first dataset and the collection of all 119 patients treated with fluoropyrimidine (including S-1, 5-FU/MTX, high-dose 5-FU, and low-dose 5-FU) as the second dataset in the same way as in the previous study [14].

### Patient characteristics and clinical parameters

A summary of the patients’ characteristics from the two datasets is shown in Additional file 1: Table S1. The association of genetic or clinical parameters with chemotherapeutic response was examined using Fisher’s exact test. Chemotherapeutic responses (complete response: CR, partial response: PR, no change: NC, progressive disease: PD) were evaluated using RECIST. We defined two groups: “CR + PR” (CR or PR) and “NC + PD” (NC or PD). Grading of clinical test values was defined using National Cancer Institute - Common Toxicity Criteria (NCI-CTC Version 2.0).

### Statistical analyses

Patients’ genotype data and clinical parameters were statistically analyzed by R packages (version 3.1.2) (http://www.r-project.org/). Further detailed theories and algorithms are shown in Additional file 2.

## Results

### Identification of rs2867461 in *ANXA3*

**Fig. 1:**
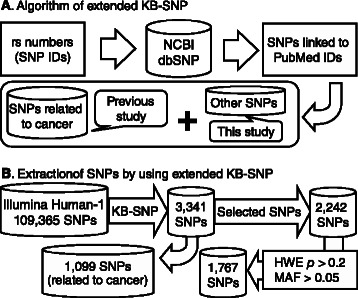
Extraction of candidate SNPs by an extended KB-SNP. We performed extended KB-SNP to identify novel candidate SNPs related to chemotherapy response. **a** SNPs linked to any PubMed IDs were extracted and the SNPs related to cancer were removed, as we had already analyzed SNPs related to cancer in the previous study. **b** A total of 1,767 SNPs were extracted from 109,365 SNPs by the extended KB-SNP and the basic filtering in the present study

We reanalyzed hypothesis-free genomic data from gastric cancer patients treated with fluoropyrimidine by applying applied our combined method with extended knowledge as described in our previous study [14], as shown in Fig. 1. Using this approach, we extracted rs2867461 in *ANXA3* as another candidate SNP related to chemotherapeutic response. Further detailed analyses and the procedure are shown in Additional file 3.

### Comparison of the models based on rs2867461 in *ANXA3*

**Fig. 2:**
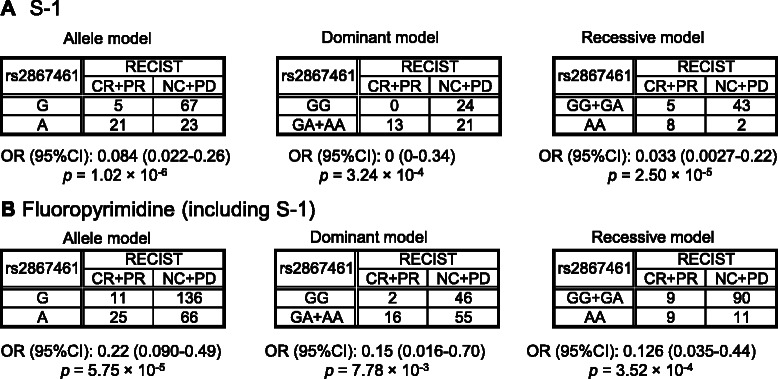
Contingency tables for rs2867461 in *ANXA3* for each model using each dataset. **a** S-1-treated gastric cancer patients (first dataset). **b** Fluoropyrimidine (including S-1)-treated gastric cancer patients (second dataset). *P* values were calculated using Fisher’s exact test. OR: odds ratio, CI: confidence interval, RECIST: Response Evaluation Criteria in Solid Tumors, CR: complete response, PR: partial response, NC: no change, PD: progressive disease

We analyzed not only an allele model, but also dominant and recessive models of rs2867461 in *ANXA3* in the first (S1-treated gastric cancer patients) and second datasets (fluoropyrimidine-treated gastric cancer patients; Fig. 2). Figure 2a shows that in the first dataset the *p* value of the allele model was the lowest (*p* = 1.02 × 10^−6^, OR = 0.084), and the *p* value of the recessive model (*p* = 2.50 × 10^−5^, OR = 0.033) was lower than the *p* value of the dominant model (*p* = 3.24 × 10^−4^, OR = 0). Similarly, Fig. 2b shows that in the second dataset the *p* value of the allele model was also the lowest (*p* = 5.75 × 10^−5^, OR = 0.22), and the *p* value of the recessive model (*p* = 3.52 × 10^−4^, OR = 0.13) was lower than the *p* value of the dominant model (*p* = 7.78 × 10^−4^, OR = 0.15). Therefore, the recessive model is the best model for rs2867461 in *ANXA3*. To evaluate combination effects of multiple factors, the proportional odds model was used to construct multiple logistic regression models.

### Selection of a model based on rs2867461 in *ANXA3* and construction of multiple regression models

**Fig. 3:**
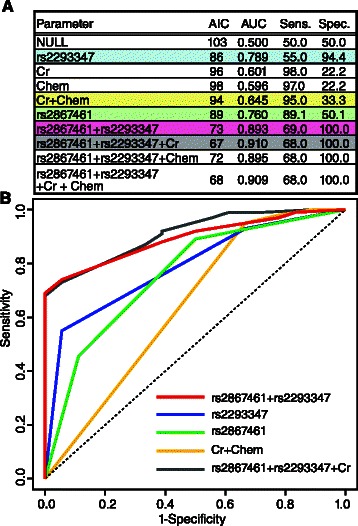
Comparison of AIC, AUC, and ROC curves between logistic regression models. **a** Parameters used for each model. **b** ROC curves for the following models: rs2293347, rs2867461, Cr + Chem, rs2867461 + rs2293347, and rs2867461 + rs2293347 + Cr. ROC: receiver operating characteristic, AUC: area under the ROC curve, NULL: model without any parameters. Each genetic factor indicates proportional odds model, AIC: Akaike’s information criterion, Sens.: sensitivity (%), Spec: specificity (%), Chem: a history of chemotherapy, Cr: grade of creatinine

We compared AICs and AUCs between 10 models: NULL (without parameters), rs2293347 (genotype of rs2293347 in *EGFR*), Cr (grade of creatinine), Chem (a history of chemotherapy), rs2867461 (the genotype of rs2867461 in *ANXA3*), rs2867461 + rs2293347, rs2867461 + rs2293347 + Cr, rs2867461 + rs2293347 + Chem, and rs2867461 + rs2293347 + Cr + Chem model (Fig. 3a). ROC curves for the five logistic regression models, Cr + Chem, rs2867461, rs2293347, rs2867461 + rs2293347, and rs2867461 + rs2293347 + Cr, are shown in Fig. 3b. All models performed better than the NULL model, although the Cr + Chem model was better than either Cr or Chem alone, and the rs2293347 and rs2867461 models performed better than the Cr + Chem model, as shown in Fig. 3a and b. Finally, the rs2867461 + rs2293347 + Cr model had the lowest AIC among the 10 models tested. Although the rs2867461 + rs2293347 + Cr model gave the best results, the best cutoff value was at a sensitivity of 68.0 % and specificity of 100.0 %, with performance depending on only rs2867461 + rs2293347, as shown in Fig. 3b. Therefore, we selected the rs2867461 + rs2293347 model as the best model in the present study, and the best cutoff value was found at a sensitivity of 69.0 % and specificity of 100.0 %. The integrated genetic factor consisting of rs2867461 and rs2293347 is a possible predictive factor of efficacy of treatment in fluoropyrimidine-treated gastric cancer patients.

### The integrated predictive index based on two polymorphisms in *EGFR* and *ANXA3*

**Fig. 4:**
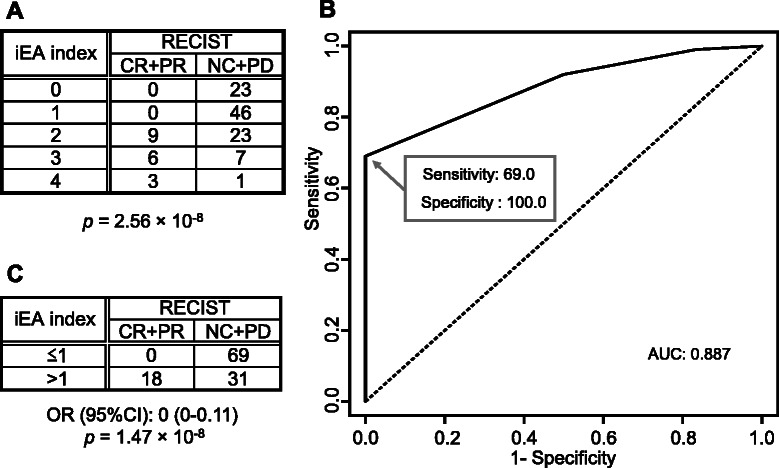
Contingency tables for integrated predictive index using polymorphisms in *EGFR* and *ANXA3* and ROC curve. **a** Contingency table for the iEA index. **b** ROC curve for the iEA index. **c** The combined contingency table for the iEA index. Abbreviations are the same as defined in Figs. 2 and 3

To define a novel predictive factor consisting of two polymorphisms in *EGFR* and *ANXA3*, we defined the total number of minor alleles of rs2293347 and rs2867461 as an integrated predictive index based on *EGFR* and *ANXA3* (iEA index). Contingency tables and the ROC curve for this novel predictive factor, iEA, are shown in Fig. 4. This figure shows that the *p* value of iEA was 2.56 × 10^−8^ by Fisher’s exact test, and a higher iEA was correlated with a formula for the better response rate (RR): ((CR + PR)/(CR + PR + NC + PD)). For example, RR = 0 % (iEA = 0 or 1), 28.1 % (iEA = 2), 46.2 % (iEA = 3), and 75.0 % (iEA = 4). Figure 4b shows that the ROC curve for the regression model based on iEA is approximately the same as the ROC for the rs2867461 + rs2293347 model. We constructed a 2 × 2 contingency table by combining contingency tables of iEA, as shown in Fig. 4c. Figure 4c shows that the *p* value of iEA was 1.47 × 10^−8^ by Fisher’s exact test. These results suggested that iEA may be an important predictive factor of response rate in fluoropyrimidine-treated gastric cancer patients. Nonetheless, clinical utility of iEA needs to be validated in future studies.

## Discussion

In the previous study, we extracted RS numbers (SNP IDs) related to cancer using a combination of National Center for Biotechnology Information (NCBI) dbSNP and NCBI PubMed [14]. In the present study, we extracted all SNP numbers linked to PubMed IDs on the basis of dbSNP but excluded SNPs related to cancer, as we had already analyzed SNPs related to cancer in the previous study. However, among these SNPs not directly related to cancer, the SNPs could still be indirectly related to cancer, as they may be involved in cellular differentiation, apoptosis, drug metabolism, transporter and immune system processes. Thus, this information may be potentially useful. Therefore, we used information of SNPs linked to any function except for cancer in the present study. Furthermore, Illumina Human-1 BeadChip is one of the most preliminary types of arrays; their detectable SNPs are not tag SNPs and it is difficult to reduce multiple comparisons problem by constructing linkage disequilibrium blocks. Therefore, we focused on the combination of dbSNP and PubMed as the most reliable information.

An SNP extracted using the combined method, rs2867461 in *ANXA3*, was previously reported as a genetic factor associated with rheumatoid arthritis, systemic lupus erythematosus, and Graves’ disease in a Japanese population [37]. Although the relationship between cancer and rs2867461 in *ANXA3* has not been reported to date, many studies have recently been published on the association between *ANXA3* and drug resistance or chemotherapy response [38]. The annexin family is a well-known multigene family of Ca^2+^-regulated phospholipid- and membrane-binding proteins [39]. *ANXA3* is a member of the annexin family, and important functions of *ANXA3* in tumor development, metastasis, and drug resistance have been demonstrated [38]. For example, *ANXA3* overexpression was found to correlate with enhanced drug resistance in ovarian cancer, promote the development of colorectal adenocarcinoma and pancreatic carcinoma, and facilitate metastasis of lung adenocarcinoma and hepatocarcinoma. In contrast, decreased *ANXA3* expression negatively correlates with the development of prostate and renal carcinoma [38]. To identify drug resistance mechanisms, Pénzváltó et al. tested 45 cancer cell lines for sensitivity to five tyrosine kinase inhibitors targeting the ERBB/RAS pathway: sunitinib, erlotinib, lapatinib, sorafenib, and gefitinib [40]. The authors identified *ANXA3* as one of the two significant genes from microarray analysis and this finding was validated by quantitative real-time PCR. To identify key proteins related to multidrug resistance (MDR) of hepatocellular carcinoma, Tong et al*.* analyzed the 5-FU-resistant BEL7402/5-FU cell line and parental BEL7402 cells [41]. Among the highly expressed proteins in BEL7402/5-FU associated with MDR, only the expression of ANXA3 was verified using an isobaric tag for relative and absolute quantitation-coupled two-dimensional liquid chromatography tandem mass spectrometry. Furthermore, in a recent study that compared EGFR-mutated and EGFR-wild type tumors, *ANXA3* was identified as one of only four downregulated genes involved in prostate cancer progression [42]. These and other results suggest that *ANXA3* is a tyrosine phosphorylation target of *EGFR* [43] and expression of *EGFR* may generally suppress expression of *ANXA3* [42]. Therefore, high expression of *ANXA3* may confer drug resistance.

According to our previous report, the rs2293347 SNP in *EGFR* was extracted as a potential predictive factor of chemotherapeutic response in Japanese gastric cancer patients treated with fluoropyrimidine [14]. This study showed that the rs2293347GA/AA genotype was associated with a lower risk of progressive disease compared with the rs2293347GG genotype (OR = 0.048, *p* = 6.32 × 10^−5^). Recently, Mochinaga et al. reported that high expression of DPD in lung adenocarcinoma is associated with mutations in *EGFR* [44]. Several studies have demonstrated that high DPD levels result in low sensitivity to fluoropyrimidine for various cancers, such as gastric cancer [45, 46], colon cancer [47], bladder cancer [48], and breast cancer [49]. Therefore, rs2293347 might affect DPD expression related to sensitivity to fluoropyrimidine.

The rs2293347G>A polymorphism located in exon 25 of *EGFR* is a synonymous SNP (D994D), while the rs2867461G>A polymorphism is located in intron 7 of *ANXA3*. These polymorphisms do not change the amino acid sequence of the protein. However, if rs2867461 and rs2293347 have no function, these SNPs are possible predictive factors linked with other functional polymorphisms in *ANXA3* and *EGFR*, respectively. Therefore, rs2867461 in *ANXA3* and rs2293347 in *EGFR* are promising predictive factors that can be used for selection of chemotherapy regimens: for instance, fluoropyrimidine alone or a combination of fluoropyrimidine with EGFR-targeting agents. Further research is needed to elucidate the clinical relevance of these SNPs.

**Fig. 5:**
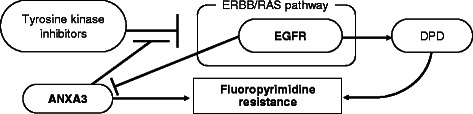
Hypothetical model of *EGFR* and *ANXA3* to fluoropyrimidine resistance in fluoropyrimidine-treated gastric cancer patients. ANXA3 overexpression confers resistance tyrosine kinase inhibitors targeting ERBB/RAS pathway. High expression of DPD is associated with mutations in EGFR. DPD is an inactivating and rate-limiting enzyme for fluoropyrimidine

As mentioned above, many studies suggest that the *EGFR* and *ANXA3* genes have relevance to fluoropyrimidine resistance and their polymorphisms have links with biological functions. Because the IntPath database is currently the most powerful tool and also the most comprehensive integrated pathway database, we first conducted pathway analysis using the IntPath database [50] to draw the genetic networks related to *EGFR* and *ANXA3*. However, we could not identify pathway information using this database. Therefore, we manually constructed a hypothetical model of relationship between *EGFR* and *ANXA3* (Fig. 5) according to the literature.

In this study, we extracted rs2867461 (which showed statistical significance according to *p* (0.0406) < 0.05) using a combination of two stages of screening and permutation testing of prefiltered SNPs for both of first and second sets. When only the first dataset was used, the *q* value calculated by the BH method was 0.00159, as shown in Additional file 4: Table S2. This *q* value is statistical significance.

Using our combined method involving two stages of screening, we identified rs2867461 as a possible genetic predictive factor. We note that our filtering methodology may have also eliminated several interesting regulatory marker SNPs that might be relevant to drug response, as shown in Fig. 1. However, the sample size of this study is not enough to identify all of these marker SNPs without omission. Therefore, we prioritized control of type I error at the cost of statistical power (type II error) in the present study. All statistical information regarding the chemotherapeutic response of gastric cancer patients treated with fluoropyrimidine (*p* < 0.05) for each SNP is shown in Additional file 5: Table S3, and the data are also provided on the website Genome Medicine Database of Japan (GeMDBJ) [51] (http://gemdbj.ncc.go.jp/omics/). These data will be useful for confirmation studies or meta-analyses in the future.

## Conclusions

In the present study, we reanalyzed hypothesis-free genomic data from gastric cancer patients treated with fluoropyrimidine by applying our combined method with extended knowledge. Using this approach, we identified rs2867461 in *ANXA3* as a candidate SNP related to response to chemotherapeutic response. The rs2867461 + rs2293347 model has greater predictive performance than clinical parameters, each single SNP (rs2867461/rs2293347), or environmental factors, and the rs2867461 + rs2293347 model had a sensitivity of 69.0 % and specificity of 100.0 %. Furthermore, in the present study, we propose a novel integrated predictive index based on the polymorphisms in *EGFR* and *ANXA3*, the iEA index. The *p* value for iEA is 1.47 × 10^−8^ by Fisher’s exact test. Collectively, iEA or the combination of rs2867461 and rs2293347 may serve as predictive factors for selecting chemotherapy regimens for the treatment of gastric cancer patients.

### Availability of supporting data

The data set supporting the results of this article is available in the Genome Medicine Database of Japan (GeMDBJ) (http://gemdbj.ncc.go.jp/omics/) with the accession number GWAS030.

## Additional files

Additional file 1: Table S1. **Selected genetic and clinical parameters of gastric cancer patients.** (XLS 33 kb)
Additional file 2: **Supplementary Methods.** (PDF 91 kb)
Additional file 3: **Supplementary Results.** (PDF 125 kb)
Additional file 4: Table S2. **Extracted SNPs with** ***q***** < 0.95 for the first dataset.** (XLS 35 kb)
Additional file 5: Table S3. **Statistical information about the chemotherapeutic response of gastric cancer patients treated with fluoropyrimidine** **(*****p*** **< 0.05).** (XLS 772 kb)
