# Supplementary material for: Construction of possible integrated predictive index based on EGFR and ANXA3 polymorphisms for chemotherapy response in fluoropyrimidine-treated Japanese gastric cancer patients using a bioinformatic method
Source: BMC Cancer. 2015 Oct 16;15:718. doi: 10.1186/s12885-015-1721-z (PMC4609065; doi:10.1186/s12885-015-1721-z)
Supplement: Additional file 2: — Supplementary Methods. (PDF 91 kb) [file 12885_2015_1721_MOESM2_ESM.pdf]

Supplementary Methods

Fisher's exact test This is a statistical test that is used to determine nonrandom associations between 2 categorical variables [1]. Fisher's exact test is similar to the chi-squared test. If the sample size is large, the chi-squared test can be used successfully, but significance values from the chi-squared test are only approximated. Fisher's exact test is a statistical test that is used to analyze contingency tables when the sample size is small [1]. We used Fisher's exact test in the present study. The odds ratio (OR) is defined as $a \times d/(b \times c)$, where a is the number of NC or PD patients with a minor allele, b is the number of CR or PR patients with a minor allele, c is the number of NC or PD patients with a major allele, and d is the number of CR or PR patients with a major allele. The null hypothesis for Fisher's exact test is OR = 1.

The permutation test The permutation test theory evolved from the works of Fisher and Pitman in the 1930s [2]. In this study, p values of multiple-comparison analyses were adjusted by applying the permutation test to two stages of screening. The case–control (or phenotype) labels were randomly shuffled for the two screening stages, and p values were calculated using Fisher's exact test. The lowest p value was selected for the randomized data. This procedure was repeated 100,000 times. Exact p values for the permutation test were calculated based on the distribution of the lowest p values.

Multiple testing correction Bonferroni correction is a method used to address the problem of multiple comparisons (also known as the multiple testing problem). It is considered the simplest and most conservative method to control the family-wise error rate (FWER). Alternatively, false discovery rate (FDR) controlling procedures, such as the Benjamini-Hochberg (BH) method [3], are more powerful (i.e., less conservative) than the FWER procedures at the cost of increasing false positives within the rejected hypothesis. In the present study, the BH method was used to calculate the q value. The q value is defined as

an FDR analog of the *p* value

Akaike information criterion (AIC) The AIC is a measure of the relative goodness of fit of a statistical model [4]. A smaller AIC indicates a better fit when comparing fitted objects. The AIC is defined according to the formula -2 × (log likelihood) + (2 × n_{par}), where n_{par} is the number of parameters in the fitted model, and the log likelihood value [5] was obtained from the logistic regression model.

The receiver operating characteristic (ROC) ROC is a graphical plot which illustrates the performance of a binary classifier system as its discrimination threshold is varied. It is built by plotting sensitivity (the number of true positive results divided by the number of true positive samples) against (1 minus specificity) at various threshold settings. Specificity is the number of true negative results divided by the number of true negative samples. The area under the curve (AUC) of a ROC curve is an indicator representing expected performance. A higher AUC is more desirable, with a value of 1.00 denoting perfect performance (sensitivity and specificity are both 100%), while a value of 0.50 indicates random performance.

References

1. Fisher RA: **On the interpretation of $\chi 2$ from contingency tables, and the calculation of P**. *J Roy Statistical Society* 1922, **85**(1):87-94.
2. Pitman EJG: **Significance tests which may be applied to samples from any population. Part III. The analysis of variance test**. *Biometrika* 1938, **29**:322-335.
3. Benjamini Y, Hochberg Y: **Controlling the false discovery rate: a practical and powerful approach to multiple testing**. *J R Statist Soc serB* 1995, **57**(1):298-300.
4. Akaike H: **A new look at the statistical model identification**. *IEEE T Automat Contr* 1974, **19**(6):716-723.
5. Sakamoto Y, Ishiguro M, Kitagawa G: **Akaike Information Criterion Statistics**. Dordrecht: Reidel Publishing Company; 1986.
