# Supplementary material for: Construction of possible integrated predictive index based on EGFR and ANXA3 polymorphisms for chemotherapy response in fluoropyrimidine-treated Japanese gastric cancer patients using a bioinformatic method
Source: BMC Cancer. 2015 Oct 16;15:718. doi: 10.1186/s12885-015-1721-z (PMC4609065; doi:10.1186/s12885-015-1721-z)
Supplement: Additional file 3: — Supplementary Results. (PDF 125 kb) [file 12885_2015_1721_MOESM3_ESM.pdf]

Supplementary Results

Associations between the chemotherapeutic response and genetic or clinical parameters. The possible genetic or clinical factors that were associated with a chemotherapeutic response were extracted previously using Fisher's exact test [1]. In that study, the genotype rs2293347 (in *EGFR*), a history of chemotherapy (Chem), and the level of creatinine (Cr; p = 0.0691) were selected as such factors (Supplementary Table S1). These genetic and clinical factors should be evaluated during construction of a multifactorial diagnostic model.

Extraction of candidate SNPs using the combined method consisting of the knowledge-based algorithm, two stages of screening, and a permutation test. We applied the extended knowledge-based algorithm to hypothesis-free genomic data from gastric cancer patients as shown in Fig. 1. Figure 1A shows an outline of the extended knowledge-based algorithm for identification of candidate SNPs (KB-SNP).

In total, 3,341 SNPs linked to PubMed IDs were extracted from the dataset of 109,365 SNPs by means of KB-SNP (Fig. 1B). Among the extracted SNPs, we were interested in the SNPs implicated in cancer in another study [1]. In the present study, we focused on the remaining SNPs (no association with cancer). Furthermore, as a basic filtering strategy, we excluded SNPs with a p-value ≤ 0.2 in the Hardy-Weinberg equilibrium (HWE) or with minor allele frequency (MAF) ≤ 0.05. The resulting 1,767 SNPs were used in the association study.

We analyzed 58 patients treated with S-1 as the first dataset during first-stage screening in the association study (Fig. S1A). SNPs with $p < 0.005$ (Fisher's exact test for the allele model) were extracted. Eight SNPs had a q-value < 0.95 (Supplementary Table S2). At the second stage of the screening, 119 patients treated with fluoropyrimidine (including 58 patients treated with S-1) were analyzed to validate these eight SNPs. Adjustment of the calculated p-value involved the permutation test for these two stages of screening

(Supplementary Table S2 and Fig. S1B). Only rs2867461 (in *ANXA3)* was statistically significant ($p < 0.05$ [= 0.0406]) after multiple testing correction based on the permutation test. Rs2867461 was a frequently occurring variant (MAF = 0.382). We assessed performance of rs2867461 via model construction.

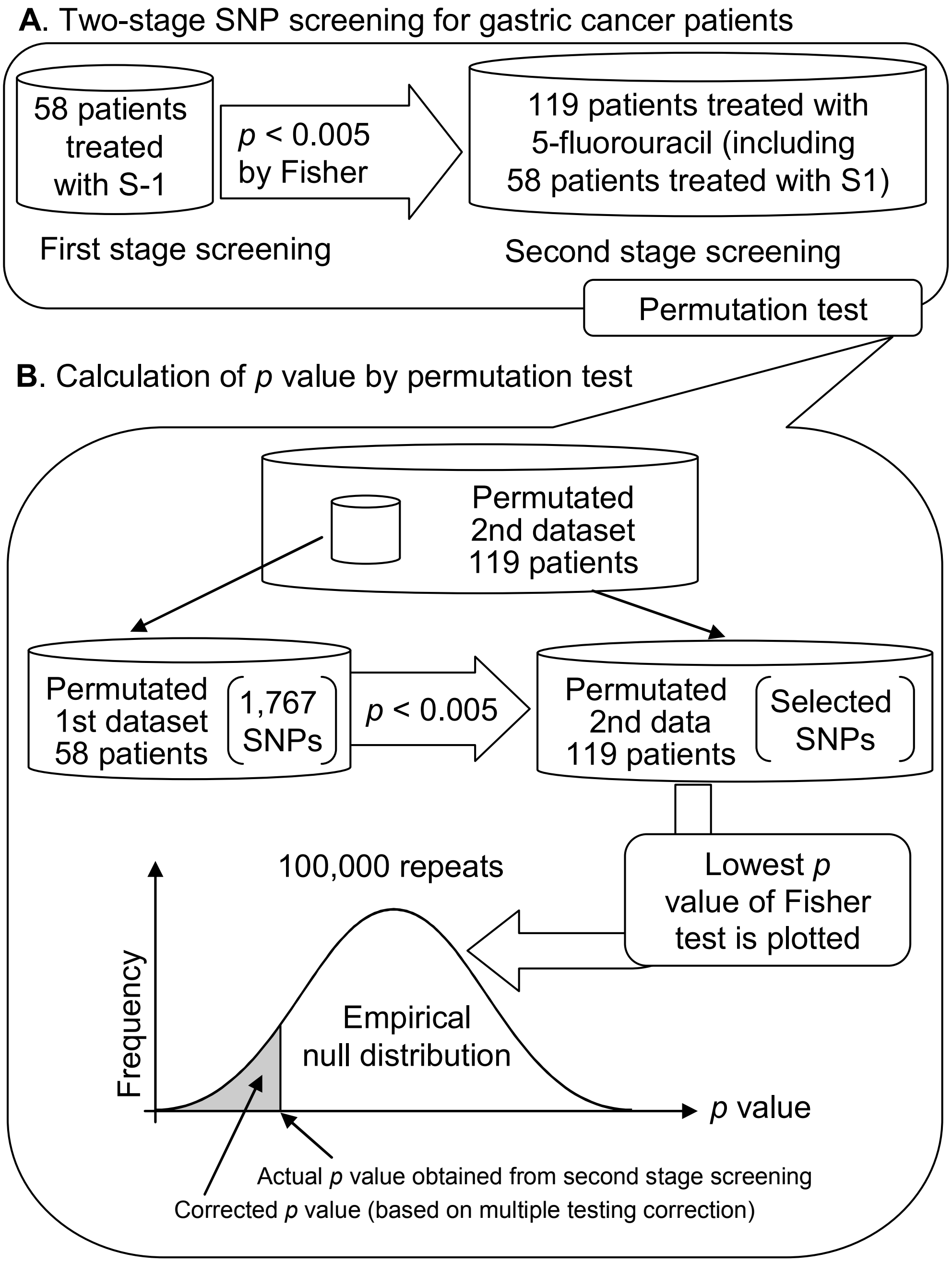

Fig. S1. An outline of two stages of screening based on the permutation test for calculation of adjusted *p*-values in the analysis of gastric cancer. (A) The two-stage screening of patients with gastric cancer for SNPs. (B) Calculation of the *p*-value using the permutation test based on the two stages of screening. The second dataset (which includes the first dataset) was permuted. From the permuted second dataset, we extracted the permuted version of the first dataset. Using Fisher's exact test and the Benjamini–Hochberg (BH) method, we selected SNPs with $p < 0.005$ in the first dataset from 1,767 SNPs. From the resulting SNPs, we selected those with the lowest *p*-value (according to Fisher's exact test for the second dataset). This procedure was repeated 100,000 times, and an empirical null distribution was constructed. Using this distribution, we converted the actual *p*-value obtained from the second stage of the screening to an adjusted *p* value (based on multiple testing correction). At the screening steps, for each SNP, we used allele models.

References

1. Takahashi H, Kaniwa N, Saito Y, Sai K, Hamaguchi T, Shirao K, Shimada Y, Matsumura Y, Ohtsu A, Yoshino T *et al*: **Identification of a candidate single-nucleotide polymorphism related to chemotherapeutic response through a combination of knowledge-based algorithm and hypothesis-free genomic data**. *J Biosci Bioeng* 2013, **116**(6):768-773.
